# When Terror and War Supersede Cancer: A Case Study of a Ukrainian Cancer Survivor and Application of Trauma-Informed Care

**DOI:** 10.1007/s10880-025-10091-3

**Published:** 2025-08-08

**Authors:** Amy Siston, Maggie Collins

**Affiliations:** 1 Psychiatry and Behavioral Neuroscience, University of Chicago Biological Sciences Division, Chicago, IL USA; 2 School of Medicine and Public Health, Department of Psychiatry, University of Wisconsin Madison, Madison, WI USA

**Keywords:** Ethnic and racial minorities, Vulnerable populations, Trauma, Psychosocial oncology, Case study

## Abstract

This case study describes the implementation of trauma-informed and culturally informed cancer care, for a 40-year-old Ukrainian female with recurrent follicular lymphoma, displaced to the United States in the Spring of 2022 following the Russian invasion of Ukraine. The patient was referred to the psychosocial oncology service at an NCI-designated Comprehensive Cancer Center for evaluation and treatment of depression and anxiety. Stressors, along with the chronic trauma of fearing for her family’s safety and the stress of being displaced from Ukraine, compounded her emotional burden. A modified form of trauma-informed care adapted for use with refugee and immigrant populations was applied, in addition to evidence-based treatments including Acceptance and Commitment Therapy, Cognitive Processing Therapy, and other cognitive behavioral techniques. At the conclusion of therapy, significant improvements were reported across physical function, social engagement, and emotional well-being. This case highlights the importance of flexibility in treatment modalities and the need to consider that the trauma of an ongoing and unpredictable war may require more therapeutic support than the experience of cancer.

## Introduction

The Russian invasion of Ukraine that began in February 2022 has led to a refugee crisis, specifically surrounding an increased need for the implementation of mental health care for anxiety, depression, and post-traumatic stress disorder (PTSD) (Javanbakht, 2022). The invasion has cumulatively displaced millions of Ukrainians from their homes to other countries (BBC, 2022). Ukrainians on the cancer care continuum have been faced with additional risk for disruption in cancer care through displacement. Little research has been conducted to explore the psychological impact of displaced individuals and refugees from Ukraine in the United States, let alone examining the experiences of those pursuing cancer treatment. Given the existing gaps in literature, we relied on several guiding frameworks and interventions to inform the psychological treatment presented in this case study.

First, we utilized Ecological Systems Theory in the context of war to conceptualize the unique systemic challenges the client faced during cancer treatment and to inform psychosocial treatment planning (Piankivska, 2022). Specifically, this theory helped to assess social support across three different levels (microsystem, mesosystem, and macrosystem). Ecological Systems Theory, initially coined by Bronfenbrenner (1977), posits that individual experiences are nested within larger structures and, therefore, the individual exists in the context of the systems surrounding them. Piankivska (2022) discussed the purpose of each level, as it relates to social support in the context of war. Social support in the microsystem consists of relatives, friends, neighbors, and colleagues. It is meant to help stabilize one’s psycho-emotional state and, in the context of war, helps to maintain a sense of one’s own safety. Social support in the mesosystem consists of small groups, hospitals, and religious organizations. It is meant to restore psychological well-being by means of community and aims to aid in adjustment to social changes and preservation of self-integrity. Social support in the macrosystem consists of society, culture, and media. It is meant to decrease resistance to recovery, serves as a measure of mental health, and is an effective principle for interventions in the early and intermediate stages after trauma. It also inspires growth, self-development, and social activity, providing protective factors through faith, traditions, and values.

Second, we utilized a multi-step framework recommended for trauma-informed care (TIC) with refugees (Miller et al., 2019). Miller’s framework emphasizes key elements of TIC: strengths-based approach, immigrant-friendly healthcare environment, trusting relationships within healthcare environment, permission to discuss difficult subjects, recognize and treat trauma appropriately, multigenerational approach to care, knowledge of local/trustworthy resources, and patient advocacy. Although Miller’s framework was originally developed for refugee children, we chose to apply it in this adult case study due to its strong emphasis on culturally responsive care, including immigrant-friendly healthcare environments and community-based strengths. While our initial approach was guided by Miller’s work, we later found that Butler et al. (2011) offered complementary TIC principles for refugees and immigrants (safety, trustworthiness, choice and control, collaboration, and empowerment) which further support the applicability of Miller’s framework to adult populations.

Third, to complement the trauma-informed framework, we incorporated findings from efficacy studies on psychosocial interventions for refugees and cancer patients (Gordon et al., 1980; Jacobsen & Jim, 2008; Rizzi et al., 2023; Turrini et al., 2019). War trauma and related stressors cause lasting physical and psychological distress. Factors like mental illness, displaced relatives, and being in an unfamiliar environment affect the severity of psychological issues. Adaptive coping strategies, social support, faith, and cultural resilience are crucial for managing these difficulties (Rizzi et al., 2023). Jacobsen and Jim’s (2008) meta-analysis highlights effective interventions for depression and anxiety in cancer populations, including psychoeducation, relaxation techniques, and supportive and cognitive behavioral therapies. Additionally, there is evidence to support the use of both Acceptance and Commitment Therapy (ACT) in psychosocial oncology (PSO) (Arch et al., 2021), with benefits demonstrated in emotional well-being, quality of life, and psychological flexibility (Gonzalez-Fernandez & Fernandez-Rodriguez, 2019) and Cognitive Processing Therapy (CPT) in trauma (Schulz et al., 2006).

## Case Presentation

### Demographic and Medical Background

*Olena* (a pseudonym) is a 40-year-old, married, female, who was born and raised in Ukraine. She has three children from her first marriage and has been married to her current spouse for five years. Olena was initially diagnosed with follicular lymphoma in 2020 and treated with standard chemotherapy (R-CHOP) outside of the United States. Olena was disease free until recurrence two years later. At that time, she had moved to the United States rather than return to Ukraine and began a second round of chemotherapy, achieving a partial response. This led to the decision to proceed with autologous stem cell transplant (ASCT) in 2022. At Day 100, Olena’s disease showed progression, and CAR T-cell therapy (CAR T) was recommended.

### Clinical Context and Presenting Concerns

Olena was referred for evaluation of anxiety and depression to a PSO service at an NCI-designated Comprehensive Cancer Center (CCC). The initial evaluation and all following appointments were conducted via telehealth due to concerns associated with COVID-19 during the appointment periods. The CCC utilizes a HIPAA-compliant platform for telehealth appointments. The attending clinical psychologist, advanced psychology trainee with trauma certification, and a professional interpreter were present for most appointments; when the interpreter was not available, Google Translate was utilized. While Google Translate is not an ideal tool for language translation in therapy, the clinicians applied ethical decision-making to weigh the pros and cons of using it when an interpreter did not arrive as planned, versus the alternative of not see the patient at all. Not seeing the patient due to circumstances beyond her control would have disrupted her care and been less ethical considering her situation.

At initial diagnostic evaluation, Olena endorsed symptoms of depression and anxiety consistent with an adjustment disorder with mixed anxiety and depressed mood. Her scores on standardized assessment of anxiety and depression (translated into Ukrainian) support this diagnosis. Olena's symptoms of sadness, worry, and guilt were primarily related to the distress she felt for her family, particularly her children, one of whom was serving as a soldier in the war in Ukraine. However, her cancer diagnosis and treatment were of secondary concern, as she often stated that she “did not worry about her health.” Several significant stressors contributed to Olena’s distress, including difficulty with acclimating to life in the United States, limited social and emotional support, social isolation due to COVID-19, decreased independence, and an inability to seek employment due to her health. Additionally, her reliance on a language interpreter for all medical appointments and minimal English skills added further strain. These stressors, along with the chronic trauma of fearing for her family’s safety and the identified stress of displacement, compounded her emotional burden.

While the providers continued with ongoing clinical assessment to consider other diagnoses, Olena’s presentation suggested that she was coping with chronic trauma and stress. This manifested more clearly as adjustment disorder with mixed anxiety and depressed mood rather than PTSD or another trauma- and stressor-related disorder. Olena endorsed PTSD-related symptoms, such as negative alterations in mood and cognition (guilt related to her family’s suffering), indirect exposure to war trauma via media, and communication with her family. However, she did not exhibit hallmark PTSD symptoms like intrusive thoughts, avoidance, or hyperarousal. Instead, her guilt was primarily triggered by positive experiences, such as seeing holiday decorations, which are not consistent with the trauma-related cues typically associated with PTSD.

## Methods

### Assessment

Olena completed self-report measures of anxiety and depression at initial diagnostic evaluation. Her scores on the Generalized Anxiety Disorder-7 (Kessler et al., 2005) and Patient Health Questionnaire-9 (Kroenke et al., 2001) (both translated into Ukrainian) indicated mild anxiety and depressive symptoms. Both measures are commonly used for distress screening in cancer care (Lazenby et al., 2015) and are effective across diverse populations (Mills et al., 2014).

Although it would have been ideal to administer assessment measures at regular intervals beyond the initial evaluation, this was not feasible. Olena had a medical proxy who received all communication and materials, including assessment measures, and then relayed them to the patient. This process prevented us from ensuring that Olena completed the measures independently and in a manner that protected her privacy. We were also concerned that completing the assessment measures during therapy sessions with the interpreter might be counterproductive to fostering Olena’s independence. Given these constraints, we assessed Olena’s psychological functioning through clinical evaluation, focusing on mood, affect, motivation, and self-reported functioning, social engagement, and subjective experiences.

### Course and Content of Therapy

Olena’s psychosocial treatment included a total of 30, 40–60-min telehealth psychotherapy sessions.

#### Sessions 1–5

Following the initial evaluation, providers collaborated with Olena to establish treatment goals, emphasizing three core areas: (1) building a local support network by connecting her with immigrant communities, cancer support organizations, and religious resources; (2) encouraging behavioral activation through structured daily routines and physical activity; and (3) promoting emotional expression through journaling. These elements were designed to foster a sense of agency and connection, which were vital given Olena’s experience of displacement and ongoing trauma.

Early sessions focused on helping Olena acclimate to the therapy format, which included a professional Ukrainian interpreter, attending clinical psychologist and advanced psychology trainee. Providers prioritized building trust to ensure that Olena felt supported in this new therapeutic environment. This was important as she prepared for ASCT while navigating the stress of displacement and the emotional strain of her family’s continued danger.

In later sessions, Olena shared the profound emotional toll of staying connected to the war through family updates and media. Being in the United States while her loved ones remained in danger left her feeling helpless, consumed by guilt, and unable to prioritize her own well-being. Her sense of powerlessness, compounded by her medical treatment and the upheaval in Ukraine, created a complex dynamic in which self-care felt both urgent and elusive.

Providers integrated ACT and CPT to address the complexity of Olena’s emotional experience. ACT helped Olena acknowledge painful emotions like guilt and helplessness while supporting her in making value-based choices, such as staying well for her family and fostering meaningful connections in the United States. CPT complemented this work by providing tools to identify and reframe distressing beliefs, including the idea that she was “selfish” for seeking medical care in the United States. Her psychological distress was compounded by the constant juxtaposition of her desire to care for her family in Ukraine with the necessity of caring for her medical needs in a new and unfamiliar context.

Providers monitored Olena’s progress through reflective dialogue, exploring her emotional state, and evolving thoughts about guilt and responsibility. They monitored behavioral activation through her self-reported physical activity and engagement in social activities. Journaling served as a therapeutic bridge, both as a method of expression and a lens into her evolving insight and resilience.

The interventions and rapport-building strategies used at the outset of treatment aligned with TIC models for refugee populations (Butler et al., 2011; Miller et al., 2019). Providers built trust by asking permission before introducing difficult topics, especially those tied to Olena’s experience of displacement. They compiled a list of local resources tailored to her medical and cultural identity, including the American Cancer Society, Ukrainian refugees support groups, and churches with rich Ukrainian culture to reduce her isolation and provide a foundation of cultural understanding. A list of local Ukrainian-speaking therapists was also provided to support care in her native language. These steps ensured that Olena’s care environment was culturally responsive and immigrant-friendly and aligned with recommendations by Miller et al. (2019) for creating a sense of cultural safety in therapy. Olena chose to continue treatment with the team at the CCC, emphasizing the importance of the established therapeutic rapport and continuity of care integrated with her medical treatment.

Providers assessed Olena’s microsystem support. She identified her American partner as a consistent source of instrumental support, helping with telehealth navigation, basic translation, and organizing medical logistics, but described emotional support as inconsistent, adding to her emotional strain and complicating her ability to manage both physical health and emotional well-being. She also identified several members of her care team, including a Ukrainian-identified nurse, as pivotal in supporting her sense of belonging and transition to life in the United States, highlighting their roles within her mesosystem. On a broader macrosystem level, she described the visible outpouring of support for Ukrainian people in the United States (evident in Ukrainian flags, fundraising, and local advocacy) as a meaningful source of cultural recognition and validation during an otherwise painful time.

#### Sessions 6–16

Each consecutive therapy session began with a structured check-in to assess Olena’s physical health, including updates on her ASCT recovery. Olena was experiencing significant fatigue, which often impacted her energy levels. Despite the physical toll of recovery, she consistently reported that the emotional burden of the war surpassed her medical concerns. She frequently described feeling profoundly lonely and anxious (loneliness rooted in her decision to shield her family from the severity of her illness, and anxiety driven by fears for her loved ones’ safety). Olena’s emotional distress was compounded by a deep sense of collective trauma. She reflected on her community’s suffering and wrestled with internal conflict: while she felt intense anger toward Russia, her Christian faith, which discourages hatred and promotes forgiveness, left her uncertain about how to reconcile these emotions.

Early in treatment, providers encouraged journaling as a strategy to help Olena process her thoughts and feelings. However, she quickly shared that journaling created intense feelings of “self-pity,” which felt unbearable and pushed her beyond her emotional limits. Drawing on Miller et al. (2019) “window of tolerance” for trauma survivors, providers recognized this technique was emotionally dysregulating. In response, they revised the treatment plan to emphasize self-care routines focused on sleep, nutrition, activity, and social connection to strengthen Olena’s emotional regulation and stability.

Given Olena’s medical fatigue and emotional overwhelm, these routines were adjusted to ensure they were manageable and did not exacerbate her stress. Providers encouraged small, attainable daily goals that prioritized physical, emotional, and spiritual health, emphasizing the importance of incremental progress.

To address Olena’s distress, providers applied ACT to help her create space for emotions like anger and guilt without becoming entangled in them. ACT allowed Olena to explore how her anger could coexist with her values of compassion and forgiveness, which were central to her Christian faith. Complementary use of CPT supported this work by identifying and reframing cognitive distortions that intensified shame or emotional suppression.

Progress was measured by evaluating Olena’s ability to access, tolerate, and regulate her emotions through strategies adapted to her needs such as modified journaling, expressive dialogue in session, and self-reflective practices rooted in compassion. Providers monitored her consistency in self-care behaviors, interpersonal functioning, and capacity to remain in her window of tolerance, allowing for flexible adjustments to coping strategies to ensure long-term healing.

Exploring Olena’s social support at the mesosystem level became increasingly relevant during this phase of treatment. Her Ukrainian church community offered both comfort and complexity. The church provided a familiar linguistic and spiritual environment where Olena could engage in prayer and faith-based reflection. Theoretically, the mesosystem plays a vital role in helping individuals adapt to adversity by reinforcing cultural values, emotional connection, and resilience (Piankivska, 2022). Research has shown that psychosocial interventions grounded in cultural identity, spiritual meaning, and community belonging can strengthen resilience in refugee populations (Rizzi et al., 2023). At the same time, Olena felt conflicted by the unspoken cultural norms of the church, which discouraged expressions of anger or hatred. To address this conflict, providers applied a strength-based approach (Miller et al., 2019), focusing on Olena’s faith as a protective factor while helping her reframe her understanding of anger. Treatment emphasized that anger could coexist with hope, faith, and love and that it was possible to express anger without compromising her values.

#### Sessions 17–24

As the war intensified in Ukraine, Olena continued to express intense feelings of anxiety, guilt, and sadness. She described moments of joy while experiencing the beauty of living in a U.S. city during the holidays and simultaneously, moments of guilt, as she questioned whether she was allowed to feel joy while her family faced daily terror. Olena also discussed the emotional strain her health placed on her partner and the additional feelings of guilt for being unable to reciprocate her partner’s loving gestures. She disclosed that she felt like their marriage was “being tested.”

Treatment included processing Olena’s grief and emotional distress with ACT techniques (e.g., present moment awareness, values exploration, cognitive defusion) that emphasized acceptance of the conflicting emotions she was experiencing, such as guilt and joy. CPT was applied to challenge and reframe distorted beliefs, such as “I am a burden to my partner.” These interventions helped Olena recognize the impact of trauma on her thought patterns and cultivate more compassionate self-perceptions.

Progress was measured subjectively by tracking Olena’s emotional responses to the self-care plan, particularly her ability to engage in activities like walking, connecting with family, and cooking, and whether these activities helped reduce emotional intensity.

Olena’s evolving relationship with her partner and adult children remained central to conceptualizations of her microsystem. Providers drew from well-established literature on cancer caregiving (Northouse et al., 2012) to guide discussions about relational strain, while attending to cultural values that shaped how Olena viewed reciprocity, independence, and support.

Consistent with trauma-informed practice (Miller et al., 2019), providers maintained a trusting therapeutic relationship and fostered an immigrant-friendly healthcare environment by staying informed with war developments, ensuring that Olena did not feel burdened with educating us on these details. Providers strived to strike a balance between being culturally knowledgeable and culturally humble, to not make assumptions outside of Olena’s lived experiences.

#### Sessions 25–30

At this point in her treatment, Olena’s medical team recommended CAR T. Olena felt ambivalent about this option and struggled to weigh the perceived benefits and drawbacks, particularly regarding whether to continue treatment in the United States or discontinue it to be with her children in Ukraine. Feelings of guilt arose from having access to cancer treatment while her family and community faced the ongoing trauma of war. This sense of guilt compounded her emotional burden, leading to all-or-nothing thinking about her options. Providers empathized with her internal struggle, working with Olena to explore more flexible perspectives, acknowledging her desire to help her family while also caring for her health.

Olena ultimately decided to pursue CAR T. She remained resilient in her approach to managing the physical side effects of cancer treatment, but the psychological distress surrounding the war and her deepening concern for her son, who was serving as a soldier, exacerbated her emotional challenges. Providers worked with Olena to explore the layers of emotional pain she carried: personal grief, helplessness regarding her health, and empathetic distress over her son’s experience.

Using ACT, providers helped Olena clarify her values as a mother and explore ways she could remain connected to her son. Sessions emphasized acceptance of what she could not control (his role in the war) while affirming her capacity to offer love and emotional presence.

CPT targeted maladaptive beliefs around self-blame and helplessness. Providers helped Olena distinguish between blame and responsibility, challenge beliefs that she was at fault for her children’s proximity to the war, and focus on empowered action within her microsystem.

Progress in therapy was tracked through a variety of measures. Providers monitored Olena’s adherence to self-care routines and her ability to maintain consistent communication with her son. They also observed her increasing capacity to hold and process grief, anxiety, and sadness without becoming emotionally dysregulated. Over time, Olena demonstrated a growing acceptance of the limits of her control, recognizing that her son’s experience in Ukraine was beyond her influence, but that her emotional presence, love, and support could still have an impact.

In line with Miller et al. (2019), providers applied a trauma-informed, two-generational approach to care that emphasizes the importance of family dynamics and relationships. Providers remained attuned to parallels and points of tension in Olena and her son’s relationship that centered on a shared theme of loss of control. We recognized that while pain was part of their experience, it could be alleviated through acceptance and relational connectedness.

### Treatment Outcome and Implications

Olena is currently disease free and remains in the United States. Progress in treatment was evident across several areas, including improvements in daily function, enhanced social interactions, and observable changes in affect, emotional expression and regulation. Olena began taking virtual English classes and actively used a translation feature on her smartphone to engage with a neighbor who accompanied her on walks. She also used this feature with medical staff, instead of relying on an interpreter. She reported feeling less emotionally distressed, which was reflected in her increased positive affect, expressions of joy, and reduced sadness and tearfulness during therapy. Despite the many challenges within Olena’s micro-, meso-, and macrosystems, Olena gradually developed clarity about what she could control and how to show up in her relationships with authenticity, compassion and care. Her journey through trauma and healing exemplified her remarkable resilience and ongoing commitment to maintaining meaningful connections despite the overwhelming health challenges she faced.

## Discussion

This case study provides promising evidence to support the use of trauma-informed and culturally informed cancer care into clinical practice. Therapists working with patients from culturally diverse backgrounds and refugees of war should remain flexible with treatment modality and consider that the trauma of an ongoing and unpredictable war may require more therapeutic support than the experience of cancer. Miller et al. (2019) research on trauma-informed practices with refugees stresses the importance of utilizing a trauma-informed lens in clinical practice, even when not completely applying TIC. By implementing the principles of TIC, therapists can better identify and treat trauma. The strength of the therapeutic relationship is one factor that is of paramount importance in meeting this treatment goal. As noted by Hanft-Robert et al. (2023), the therapist needs to be cognizant of all working relationships (i.e., therapist-interpreter and interpreter-patient) with the goal of fostering a working alliance and ultimately providing the patient with the feeling of safety. Therapists also need to consider that the inclusion of an interpreter in psychotherapy with trauma-affected refugees may be perceived with hesitancy, and if a working alliance is not established, it can negatively impact the therapeutic process (Hanft-Robert et al., 2023).

### Complicating Factors & Barriers to Care

Several complicating factors and barriers to Olena’s care emerged. Consistent access to Olena’s preferred interpreter was difficult due to the logistics involved with the interpreter service. It became clear early on that Olena felt comforted and connected with Ukrainian interpreters. Communication barriers especially when relying on Google translate may have impacted our ability to accurately conceptualize Olena’s needs. Notably, Olena was often less concerned about her cancer care than about the war and her family’s safety in Ukraine. The writers needed to be creative in terms of treatment planning, considering she was a patient within a PSO. We felt strongly that, considering her choice not to pursue outside referrals, it would have been more harmful than helpful to discontinue services solely due to the typical workflow in this PSO service. Lastly, as non-Ukrainian providers, our ability to understand some of Olena’s macro-level societal and cultural norms was limited.

### Recommendations and Follow-Up

Trauma-informed cancer care is not the same as trauma-specific treatment. Rather than following a trauma-specific protocol, it focuses on patient’s emotional response to stressors (Archer-Nanda & Dwyer, 2024) and remains adaptive and integrative due to the evolving nature of the week-to-week stressors common in PSO settings. There are unique cultural considerations while working with patients impacted by the war in Ukraine, wherein displacement trauma interferes with the cancer care experience. We recommend trauma-informed cancer care for patients such as ours to include strength-based approaches that include trust, permission, trauma response education, community resources, and advocacy. Unique cultural considerations for the therapist include balance of cultural humility and culture-specific knowledge, attention to global news and opinion and balance of problem-solving, emotional processing and support.

## Data Availability

No datasets were generated or analyzed during the current study.
